# Recent Advances in Understanding the Protein Corona of Nanoparticles and in the Formulation of “Stealthy” Nanomaterials

**DOI:** 10.3389/fbioe.2020.00166

**Published:** 2020-04-03

**Authors:** Riccardo Rampado, Sara Crotti, Paolo Caliceti, Salvatore Pucciarelli, Marco Agostini

**Affiliations:** ^1^First Surgical Clinic Section, Department of Surgical, Oncological and Gastroenterological Sciences, University of Padua, Padua, Italy; ^2^Nano-Inspired Biomedicine Laboratory, Institute of Paediatric Research—Città della Speranza, Padua, Italy; ^3^Department of Pharmaceutical and Pharmacological Sciences, University of Padua, Padua, Italy

**Keywords:** nanoparticles, theranostics, interface, protein corona, immunology, characterization, stealth, anti-fouling

## Abstract

In the last decades, the staggering progress in nanotechnology brought around a wide and heterogeneous range of nanoparticle-based platforms for the diagnosis and treatment of many diseases. Most of these systems are designed to be administered intravenously. This administration route allows the nanoparticles (NPs) to widely distribute in the body and reach deep organs without invasive techniques. When these nanovectors encounter the biological environment of systemic circulation, a dynamic interplay occurs between the circulating proteins and the NPs, themselves. The set of proteins that bind to the NP surface is referred to as the protein corona (PC). PC has a critical role in making the particles easily recognized by the innate immune system, causing their quick clearance by phagocytic cells located in organs such as the lungs, liver, and spleen. For the same reason, PC defines the immunogenicity of NPs by priming the immune response to them and, ultimately, their immunological toxicity. Furthermore, the protein corona can cause the physical destabilization and agglomeration of particles. These problems induced to consider the PC only as a biological barrier to overcome in order to achieve efficient NP-based targeting. This review will discuss the latest advances in the characterization of PC, development of stealthy NP formulations, as well as the manipulation and employment of PC as an alternative resource for prolonging NP half-life, as well as its use in diagnostic applications.

## Introduction

Nanotechnology for years held the promise of radically improving detection and treatment of many different diseases. The concept of using nanomaterials to improve the delivery of drugs and enhance the diagnosis of pathologies has driven biomedical research for decades. These efforts have brought to the development of a wide swath of nanovectors, with highly heterogeneous compositions and applications. However, despite the development of countless nanovector iterations, only a very small fraction of these platforms successfully reached the clinic (Ventola, 2017). This high attrition rate can be explained by the sub-optimal biodistribution and safety profile of NPs after administration. In fact, most of the particles are unable to reach the target and accumulate mostly in off-target organs like the liver, spleen, and lungs, due to mononuclear phagocytic system (MPC) clearance (Zhang et al., 2016).

**Figure 1:**
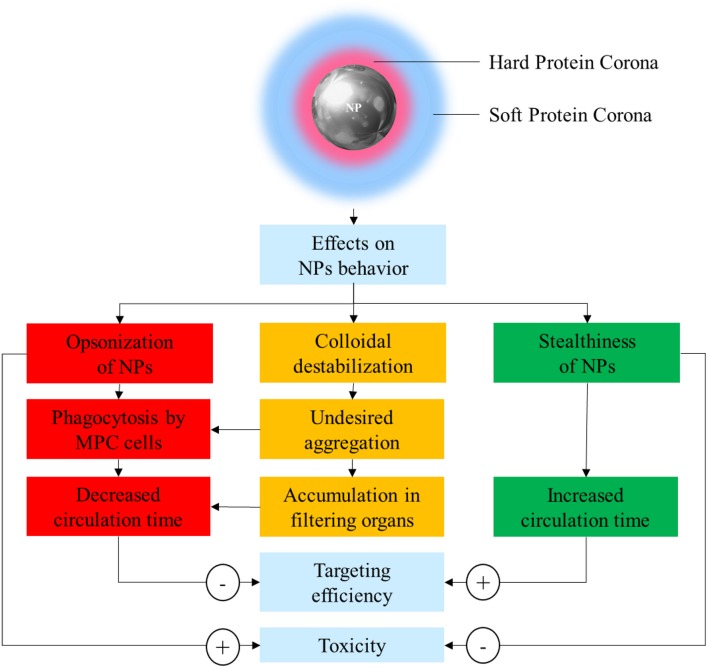
Schematic representation of the possible effects of protein corona (PC) on nanoparticle (NP) stability, safety, and pharmacokinetics.

In order to solve these issues, it is paramount to achieve a better understanding of the interaction between NPs and the biological environment they are exposed to. In the sixties, Vroman discovered that when a synthetic material, including NPs, comes in contact with any biological fluid, it becomes quickly covered by resident proteins (Vroman and Lukosevicius, 1964; Vroman et al., 1980). The array of proteins that become attached to nanovectors is collectively referred to as the protein corona (PC), and its assembly is considered the very first interaction between NPs and their biological milieu. The composition of PC is highly variable and depends on many factors including size, material, and surface charge of NPs. The assembly of this protein coating bestows NPs with a new biological identity that determines their colloidal stability, biodistribution, interactions, toxicity, and clearance (Figure 1). PC architecture is normally distinguished in a “hard” PC (HPC) in close contact and strongly interacting with the NP surface, and a more external layer of loosely and indirectly bound proteins defined as the “soft” PC (SPC). SPC is much more dynamic than HPC due to quick exchange in proteins occurring with the biological environment, making it much more elusive to isolate and characterize.

PC has been often considered a “fluid biological barrier,” something to be avoided for the nanoparticles to successfully achieve tumor targeting. This is because PC is often the prime reason for loss of NP stability, quick clearance, and potentially harmful immunologic reactions (Westmeier et al., 2016). Following this principle, the design of NPs has focused on the development of new strategies to reduce or slow PC formation. This strategy aims to improve the stability and circulation time of nanovectors, using synthetic or biological materials defined as “stealth-inducing” or “anti-fouling.” However, this simplistic understanding has been challenged and overcome, thanks to the progresses in the understanding of the roles of the individual proteins composing the PC (Schöttler et al., 2016). Indeed, deciphering and understanding the PC functions and significance is of critical importance to inform the optimal design of novel NP formulations.

In this review, we will give an insight into the composition, assembly, and analysis techniques of PC on NPs, including the latest advances in the formulation of NPs able to alter the PC formation, by either slowing or manipulating it, and we will expose limitations and future perspective of recent studies on the topic.

## Factors Influencing the Composition and Assembly of the Protein Corona

The wealth of data recently produced regarding the PC demonstrated how its composition depends on both the NPs features and the composition of the biological substrate they interact with (Tables 1, 2). This latter variable depends on inter-individual (e.g., age, gender, diet, state of health) and inter-species characteristics. These differences have very important repercussions on the toxicology of NPs, and on its evaluation during pre-clinical testing (Corbo et al., 2016, 2017b,c). As a proof of this, the inter-species dependence of PC was evidenced by a recent study on the characterization of PC composition of PEGylated silica NPs actively targeted with transferrin after incubation with either human serum (HuPC) or mice serum (MPC) (Solorio-Rodríguez et al., 2017). PC composition assessment revealed that in both human and mouse serum incubation, most of the proteins had a molecular weight (MW) between 20 and 80 kDa, and an isoelectric point (IP) between 5 and 8. However, despite this overlap between HuPC and MPC, PCs differed in their composition, and less abundant proteins presented a higher inter-species variability. Indeed, MPC presented a higher amount of proteins involved in blood coagulation, while immunoglobulins (Igs) and complement proteins mostly characterized HuPC. Such a species-specific composition of PC can justify the observed adverse effects during preclinical investigations and should be considered during NP design translation from animal models to the clinical practice.

**Table 1:** NP features influencing the formation, composition, and characteristics of the PC.

**Feature**	**Influence on NP interactions with proteins**	**References**
Size	• Larger particles have lower and offer more surface interaction for each protein. • Smaller particles have higher surface curvature. This leads to less influence on the protein's conformation.	Xu et al., 2016 Magro et al., 2019
Shape	• Shape change the mass/surface ratio of NPs. Spherical NPs (maximum mass/minimal surface) thus minimize the interactions with the environment. • Shape changes the curvature of NPs, with the above-mentioned repercussion of protein conformations.	García-Álvarez et al., 2018
Hydrophilicity/hydrophobicity	• Hydrophobic NPs interact with hydrophobic proteins through Wan der Waals or π-π interactions. • Hydrophobic surfaces could favor protein denaturation/conformational chances, by forcing to expose their hydrophobic domains. • Hydrophilic NPs interact with more charged proteins through electrostatic interactions.	Saha et al., 2016
Surface charge	• More densely charged NPs tend to have thicker and denser PCs. • Highly positively charged NPs interact very quickly and very strongly with proteins having an IP <5.5. • Highly negatively charged NPs interact mostly with proteins with an IP >5.5. • Slightly negatively charged proteins appear to have lower interactions with proteins.	Almalik et al., 2017 Partikel et al., 2019

**Table 2:** Environmental and experimental settings influencing the formation, composition, and characteristics of the PC.

**Feature**		**Influence on NP interactions with proteins**	**References**
Medium	Protein amount	• Total proteins amount in the medium affect the thickness and composition of PC.	Zhang et al., 2017 Partikel et al., 2019
	Composition	• Biofluids' origin (e.g., interstitial fluid, blood, plasma, serum) influences the PC composition. • The presence of cell culture medium can influence PC composition.	Bonvin et al., 2017 Cox et al., 2018 Ho et al., 2018 Berardi and Baldelli, 2019
	Source	• The species of animal (e.g., rat, bovine, or human) affect the PC composition. • In samples from humans, inter-individual variability (age, sex, diet, and health state) have shown to influence PCs.	Corbo et al., 2017b; Solorio-Rodríguez et al., 2017
Temperature and pH	• Temperature of incubation influences the protein diffusivity and the affinity toward NPs. • pH can influence NPs and protein surface charge and reciprocal affinity		Raoufi et al., 2018 Gorshkov et al., 2019
Time	• Following the Vroman effect, the time of incubation is a critical parameter, especially for short time points, since the protein-binding dynamics change very quickly within a few minutes of incubation.		Tenzer et al., 2013 Hadjidemetriou et al., 2016
Fluidics	• Dynamic conditions (especially PC formed after *in vivo* administration) give a much more realistic representation of the actual PC composition and are more heterogeneous. • PC conformation is less homogeneous upon dynamic conditions, leaving portion of the NPs not coated and free to interact with cells.		Hadjidemetriou et al., 2015 Hadjidemetriou et al., 2019 Pozzi et al., 2016
Isolation technique	• Centrifugation may remove loosely bound proteins from the NPs, thus providing only a rough picture of the hard PC. • Strong centrifugation could destabilize less dense NPs, such as liposomes, by mechanical stress. • A combination of size exclusion chromatography and filtration represents a good alternative to centrifugation.		Carrillo-Carrion et al., 2017

### Incubation Conditions

During *in vitro* incubation, pH and temperature conditions strongly influences the protein affinity for the NPs. Recently, Gorshkov et al. (2019) suggested that PCs consist of kinds of proteins: those that are sensitive, and those that are resistant, to temperature or pH perturbations. Even if in a restricted physiological range (from ~37 to 40°C/41°C), temperature may influence the protein diffusivity and the affinity toward NPs. Conversely, structural alterations of proteins on the NP surface can occur under the influence of pH. Environmental pH of different biological compartment spans form acidic (1.5/2 stomach or 4.5/5 in lysosomes) to neutral/slightly basic (blood and duodenum). pH influences both PC stability (*via* salt bridges and hydrogen bonding) and PC protein folding (Raoufi et al., 2018).

Composition of the PC can be quite different if NPs are exposed to the biologic fluids *in vitro* or *in vivo* (Hadjidemetriou et al., 2015). In a study, the PC of naked liposomes, PEGylated liposomes, and mucin-1 actively targeted PEGylated liposomes were assessed (Pederzoli et al., 2018). NPs were either incubated with mouse serum under agitation, in order to allow the assembly of PC *in vitro*, or liposomes were injected intravenously (IV) in CD-1 mice, and the blood was recovered after 10 min. Relevant differences have been found in the conformation of PC-composing proteins. The *in vitro* PC showed a high presence of fibrillar proteins covering homogeneously the surface liposomes. Conversely, *in vivo* assembled PC had a less homogeneous and non-fibrillar pattern. Notably, *in vivo* PC was much more diverse in its composition and showed more protein amount than the *in vitro* condition. Similarly, the study by Hadjidemetriou et al. (2019) evidenced the difference in PC composition as a function of incubation conditions. Indeed, the exposition of doxorubicin-loaded liposomes (Caelyx®) with the blood from the very same patient *ex vivo* instead *in vivo* caused a substantial modification in the overall amount of PC proteins. Moreover, in human Caelyx® PC, the main proteins found were immunoglobulins, fibrinogen, albumin, apolipoproteins, and, to a lesser extent, some complement factors. The presence of immunoglobulins and complement can be considered the major players in the small fraction of patients that develop C activation-related pseudo allergy (CARPA), although this phenomenon was not observed in the patients involved in the study. Perhaps the most important result of this study is due to the observation that the most present protein in the PC was the CS0DD006YL02 protein. Remarkably, this protein was not detected in the control plasma derived from the same patients. The appearance on the liposome's PC could be due to the high affinity of this protein for the NPs, despite its extremely low amount in the blood. This PC-mediated enrichment could make this molecule more detectable, since the high noise from heavier molecules present in the blood at higher amounts is reduced.

Static and dynamic (i.e., the flow rate) conditions were demonstrated to be critical in determining the protein composition in the PC compared even with the biologic fluid. For instance, in the study by Bonvin et al. (2017), the PC composition formed *in vitro* was investigated under different flows mimicking those present *in vivo*. Indeed, blood velocity in humans spans over three orders of magnitude (from 0.03 to 30 cm s^−1^). Although the PC is *per se* enriched in some proteins from the original biologic fluid, the increase in flow rate increases or decreases the amount of specific proteins. The proteins enriched at higher flow rates (coagulation factor V, and isoform 3 of plasma protease 3 inhibitor) interestingly were characterized by more structural flexibility, suggesting a conformational contribution to stable binding to NPs. Some proteins, however, were not influenced by the flow rate, demonstrating particularly high affinity for the NPs. By alternating the incubation with different medium (blood and then lymph, and *vice versa*), Bonvin et al. highlighted that PC composition was substantially different, depending on the order of NP incubation with blood and lymph. Moreover, especially at lower flow rates, PC retained the fingerprints of the first compartment encountered, suggesting the possibility of PC evolution across the organism, as a function of the administration route and the biological compartments encountered.

### Kinetics of PC

**Figure 2:**
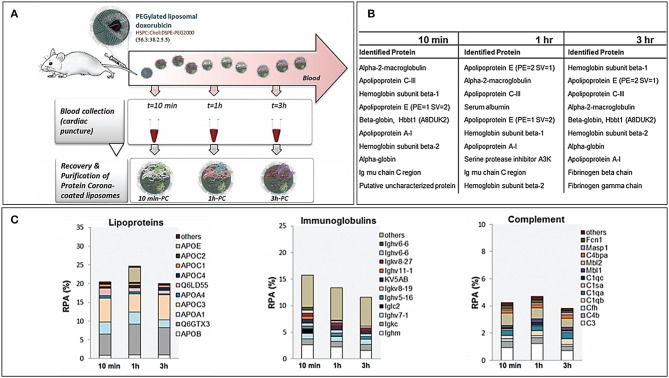
**(A)** Workflow of the study for the *in vivo* time-dependent assessment of Doxil PC composition. **(B)** Top 10 proteins for abundance in decreasing order at the three registered time points. **(C)** Composition by functional class of the Doxil® PC over time. Figure reproduced with permission from Hadjidemetriou et al. (2016) with modifications.

PC forms almost immediately onto the NPs after exposure to biological fluids, but its composition can vary over time. The assessment of PC over time allows also to get an insight of the kinetics of protein binding and its changes over time. More specifically, some proteins tend to decrease or increase over time, while others have only a transient increase (or decrease). Hadjidemetriou et al. assessed PC composition over time of PEGylated liposomes loaded with doxorubicin (Hadjidemetriou et al., 2016). In this work, liposomes were injected IV into CD mice, and blood was harvested at 10 min, 1 and 3 h after. Dynamic light scattering (DLS) and transmission electron microscopy (TEM) analyses demonstrated how PC formation occurred as soon as 10 min after administration. However, the amount of adsorbed proteins did not substantially change over time, and most of the identified proteins were constant. The number of proteins present exclusively in each single time point decreased over time, suggesting the tendency toward a late equilibrium state. Furthermore, the most abundant proteins among different time points were roughly the same, but their abundance fluctuated: at 10 min, the most abundant protein was macroglobulin, while at later time points, there was a prevalence of hemoglobin and apolipoproteins. This pattern is quite interesting, since apolipoproteins are considered as “dysopsonins,” or proteins able to improve the circulation time of NPs by preventing the binding of proteins that would otherwise enhance the clearance of NPs (such as complement and immunoglobulins) (Figure 2). These kinetic considerations have important repercussions while thinking about the NP biodistribution. Indeed, NPs reach the tumor milieu at different moments of this “PC life” and, therefore, have a partially different biological identity that could interact, at least theoretically, differently with the target cells. However, in literature, there are still a few studies that address such an elusive variable, and the relevance of PC dynamism is still to be clarified.

### NP-to-Protein Ratio

Despite PC being considered as normally responsible for NP colloidal instability, recent studies have demonstrated how the protein amount and their nature have a strong effect on NP stability. In the study by Ho et al., AuNPs were incubated with increasing concentrations of HSA, fibrinogen, immunoglobulin, and ApoA1 (Ho et al., 2018). For all these proteins, the particle aggregation had inverse proportion pattern: at lower protein concentrations, all the proteins increased NP aggregation, but at higher concentrations, the NPs appeared to be stabilized. Among the proteins, immunoglobulins, and fibrinogen induced much higher particle aggregation compared to HSA and ApoA1. This could be explained by different factors: first, immunoglobulins and fibrinogens are inherently “sticky” proteins, which function is to bind to foreign bodies and to form clots, respectively. Thus, the ability of these proteins to interact easily with many different entities could lead to their efficient interaction and agglomeration of NPs. Also, immunoglobulins and fibrinogen are much larger proteins than HSA and ApoA1, thus, interacting at the same time with more NPs by forming molecular bridges. Conversely, HSA and ApoA1 are proteins with transport function and are much smaller, thus, covering more efficiently the NP surface, preventing inter-particle interaction.

Similarly, the impact of NP-to-protein ratio in a complex medium has been assessed by a recent study by Zhang et al. (2019). NPs coated with differently charged surfactants to modulate their zeta potential (ζ) had different PC features based on their concentration in solution (range 125–1,000 μg/ml) after incubation with 5% FBS at different time points over a period of 60 min. After incubation, the negatively charged NPs changed substantially their value of ζ toward a final value around −16 mV; positively charged particles instead lost their positive charge and acquired a negative ζ. However, at the highest concentration (1,000 μg/ml) the ζ was less negative than expected, probably due to a “saturation” effect. Indeed, at such a high concentration, the total amount of proteins per particle is substantially decreased, leaving some positive charges still exposed. Despite this, there is a very interesting preliminary insight in the change of PC composition; normally, the dose of the administered NPs is such that the available proteins largely exceed the NP surface, making the use of high NPs/protein ratios somewhat unrealistic.

### NP Biodistribution/Disposition

NP fate after initial administration *in vivo* (by oral administration, IV, or subcutaneous) could follow different paths: transfer from an extracellular fluid to another, transmembrane migration, and interactions with different cell populations before arrival to their destination. In the simplest scenario, NPs are administered IV and come in contact first with blood and then with lymph before reaching their target. On the contrary, NPs administered subcutaneously follow the reverse order of exposition. When NPs reached the blood, their fate may depend on the formed PC. Although it seems to be established that a PC is highly enriched in proteins such as complement factors, immunoglobulins, and coagulation factors is indicative of NP short blood circulation time and quick removal from circulation, the actual efficiency of these proteins working as opsonins for phagocytic cells is not well-established. In the study by Saha et al. (2016) on gold nanoparticles (AuNPs), the major proteins found in PC consisted of complement proteins, immunoglobulins, apolipoproteins, coagulation proteins, and acute phase proteins. As expected, a higher uptake on macrophages was observed for formulations presenting high amounts of complement components (especially C4BPA). On the contrary, in a study by Caracciolo et al. (2015) in which PCs were enriched in opsonins, their uptake by macrophages after PC assembly was markedly reduced compared to bare NPs. This suggests that despite the composition of PC, other factors may affect the opsonization. In fact, despite the presence of opsonins in the PC, only a small fraction could remain in their functional state after binding to NPs and also be oriented in such a way that their functional domains are exposed to the outer side of the PC, which is a necessary condition to interact with cellular receptors.

Oral administration offers several advantages such as non-invasiveness, good patient compliance, and can be advantageous in delivering drugs to the GIT itself. However, this route poses several important challenges: the GIT is characterized by an extreme complexity, with different segments of this system having different pH, enzymes, cell populations, and the presence of mucus. Furthermore, several factors (intestinal flora, presence of food, GIT disorders) can alter the intestinal mobility, permeability, and microenvironment composition over time. Actually, there are very few studies regarding the GIT PC formation and composition of orally administered NP formulations (Berardi and Baldelli, 2019). This discrepancy calls for a more systematic study on how the presence of PC can influence the orally administered NP colloidal stability, drug release, and intestinal permeability.

Finally, the presence of biological barriers that cannot be easily penetrated can be a limiting factor for NP biodistribution. One of these barriers is represented by the blood–brain barrier (BBB). However, little is known about how the PC of NPs changes when they cross this hurdle. One of the few studies on this topic is provided by Cox et al. (2018). In this work, the PC composition of 3.5 nm NPs is assessed before or after their crossing of a Transwell *in vitro* model of BBB. Interestingly, the PC composition is changed dramatically upon BBB crossing because of both the different protein composition of the two media and the crossing of the BBB, itself. Indeed, NPs interacting with intracellular compartments of BBB are exposed to different microenvironments that could alter the PC. Remarkably, this cell-based mechanism appears to be the most relevant, and the PC formed after BBB crossed appeared more stable, perhaps due to the cellular removal of a portion of PC proteins, leading to a stronger binding of the residual ones. After overcoming the BBB, NPs encounter a new biological compartment populated by neuronal and specialized immune cells (the microglia). For this reason, some studies also focused on the evaluation of PEG on the NPs targeting the efficiency of different CNS cell populations. Jenkins et al. demonstrated how the use of PEG to coat magnetic NPs reduced their uptake from CNS immune cells but at the same time also reducing the uptake from neurons (Jenkins et al., 2016). These results are in line with those previously obtained and indicate *de facto* a reduction of targeting efficiency (Suk et al., 2016), creating the so-called “PEG dilemma.”

## Analysis of the Protein Corona

Many different techniques are available for the analysis of the composition and thickness of the PC. However, none of these experimental approaches is exhaustive, and thus, the combination of different analytical approaches is essential in order to characterize PCs. The following section is just an overview of the main techniques employed in the most recent studies on PC, and the interested reader is directed to specialized reviews (Carrillo-Carrion et al., 2017).

The presence and thickness of PC on NPs can be assessed using techniques such as dynamic light scattering (DLS) and transmission electron microscopy (TEM). DLS allows for the measurement of the NP hydrodynamic diameter. The increase in NP diameter after their incubation with a biological medium is normally attributed to the protein adsorption onto the NP surface. By calculating the difference between the original NP diameter and the PC–NPs diameter, the thickness of the PC may be estimated. However, the apparent increase in NP hydrodynamic diameter is several times than that of the original NP size, meaning that other mechanisms can contribute to this increase (NP agglomeration due to their colloidal destabilization, molecular bridge formation by the PC, itself). DLS allows also for the estimation of zeta potential (ζ), which is dependent on the NPs' surface charge. Normally, after NP incubation with proteins, the zeta potential tends toward a value of a few mV below zero, independent from the NPs' original charge. This ζ shift is often attributed to the protein on the surface of the NPs.

In the case of TEM, the protein corona presence is detectable directly by evidencing the presence of an electron-dense “halo” of proteins surrounding the NPs. However, this technique is not free from caveats, since the sample preparation, itself, can have effects on the PC causing protein destabilization. A relatively milder approach is to use Cryo-TEM, which allows the visualization of PC in its native state through extremely quick freezing of the sample. These techniques, however, have low throughput and cannot be used routinely on many formulations.

Besides thickness, the protein amount in PC is another parameter to evaluate. Among the simplest approaches available is simple protein quantification using colorimetric assays (Braford or BCA) that give the amount of protein in mass, but without any information regarding their composition. However, it is important to consider the possible interference of NP components on the yield of this colorimetric assay. This limitation could be overcome by creating adequate calibration lines of standard protein concentrations in the presence of particles.

A slightly more advanced technique relies on SDS-PAGE or two-dimensional gel electrophoresis (2DIGE) coupled to densitometric analysis. This analysis is easy, quick, and cheap and allows for the resolution of proteins depending on their molecular weight. The additional information this approach provides is evidencing the relative abundance of proteins at different molecular weights that can suggest qualitative changes in the PC composition. Despite some studies trying to identify a protein based only on the molecular weight, this does not provide true evidence of the protein's identity. Furthermore, SDS-PAGE is not very sensitive and provides only a rough estimation of the molecular weight. Gel-based separation allows for better confidence in single protein identification when coupled to mass spectrometry or Western blot analyses.

Mass spectrometry-based proteomic analysis is the most useful technique to obtain a confident protein identification (Capriotti et al., 2014; Carrillo-Carrion et al., 2017). Through an opportune experimental setup, it is possible to identify, quantify, and characterize (in terms of PTMs) the PC protein composition in a single analysis. Downstream bioinformatics analyses of proteomics data permit to cluster hundreds of identified proteins using different features such as molecular weight, isoelectric point, and biological function. Even if these techniques are among the most sensitive and powerful, providing high amounts of information even about proteins with low abundances composing the PC, there are some limitations and caveats that must be taken into consideration. Indeed, proteins present at very low abundance can be “masked” by the abundant ones, through different phenomena (unbalance in the tryptic digestion efficiency, in-source ion suppression, lack of precursor isolation, fragmentation, etc.). Collectively, these events may offer a skewed quantification of rare proteins. Capillary electrophoresis is another technique that allows for quick and even real-time analysis of PC formation by analyzing the change in elution rates of specific proteins in order to understand their interactions with the NPs in study. This strategy is often much quicker than gel electrophoresis, allows for the analysis of proteins in solution (and thus in their native state), and does not require the isolation of NPs from proteins before the analysis. Capillary electrophoresis can be coupled to other analytical techniques as well, including mass spectrometry, for protein identification.

When it comes to understanding the affinity of specific proteins toward NPs, highly specialized techniques are required. Among the most employed ones, isothermal titration calorimetry (ITC) allows to obtain several information and calculate the binding constant of a protein to the NPs, the protein to NP stoichiometry, and the NP surface area that each one occupies. However, this analysis does not allow for the calculation of such precise parameters for more than a single protein at a time, making its application not very easy to correlate to the complex array of molecules present in biologic fluids. Another option is based on the use of surface plasmonic resonance (SPR) probes with the immobilized proteins on their surface. These probes can change their optical proprieties depending on the adsorption of NPs on their surface and then permits to accurately measure the NP–protein interactions in real time and even under flowing conditions (Canovi et al., 2012).

The formation of the PC often forces the protein adhering onto the NP surface to change conformation in order to stabilize their binding, and some cases even cause partial protein denaturation and fibrillation. The biological relevance of this phenomenon is essential since several opsonizing proteins (such as complement factors, coagulation factors, and immunoglobulins) rely on conformational changes in order to activate their respective signaling cascades, and otherwise, some *di per se* non-immunogenic proteins may expose new epitopes that can trigger subsequent opsonization. To understand the changes in the protein structure, the most used technique is circular dichroism (CD) that permit to highlight changes in the percentage of specific structural components (e.g., alpha helices, beta sheets, or random chains) after incubation with NPs, indicating the change in protein conformation and evidences of its denaturation. More recently, conformational alterations have also been predicted and simulated *in silico*. This strategy is quite elegant and allows for a complete assessment of the chemical nature of the interaction as well as for the protein affinity and behavior on the NP surface. However, this approach is also quite difficult, and the correlation between simulated conditions of interactions and reality is not always straight forward (Lopez et al., 2017).

## Recent Advances in “Stealth” Nanoparticle Design

In the last decades, the understanding of PC as one of the primary factors contributing to NP destabilization and quick clearance after injections led to the development of many nanomaterials with the specific focus of reducing or slowing down the PC assembly, thus, improving NP biodistribution. In the present section, an overview of the latest advances regarding these materials is presented.

### Synthetic Approaches

Although PEG has been considered for many years as the gold standard of stealth-inducing materials and is still used for the production of stealth drug delivery systems (Pasut et al., 2015; Chen et al., 2018; Cui et al., 2018; Viard et al., 2018; Zhong et al., 2019), many of its limitation have recently been evidenced. First, PEG does not only hinder the interactions of NPs with phagocytic cells responsible for their quick clearance, but also reduce the efficiency of uptake by the target cells NPs are directed against (Zhang et al., 2015). Second, multiple administrations can trigger the formation of circulating anti-PEG antibodies, which can thus hinder the actual utility of this polymer in the first place and prime hypersensitivity reactions, which are a great concern for NP safety (Gref et al., 1994).

The first limitation may be overcome by a careful optimization of PEG features such as length, coating density, and overall structure, which must be considered in order to maximize its benefits. For example, for the clinically available Doxil, it was found that the optimal PEG length was 2 kDa, since it could prolong the NP circulation time while not hindering their cellular uptake by target tumor cells (Barenholz, 2012). PEG polymer can reduce drastically the adsorption of proteins on the NP surface, thus preventing relevant opsonization (Suk et al., 2016). A recent study by Naidu et al. that analyzed the PC composition of PEGylated and non-PEGylated poly (glycidyl methacrylate) (PGMA) NPs confirmed this aspect (Naidu et al., 2017). However, this polymer actively tunes the composition of the PC, itself, as evidenced by the different abundance and even the exclusive presence of certain proteins compared to the non-PEGylated NPs. This suggests that PEG can also modulate the presence of some critical proteins, such as clusterin (Saha et al., 2016), on the NP surface that could be beneficial to NP circulation and stability.

The issue of production of anti-PEG immunoglobulins as stated above is a major hurdle in the chronic and widespread use of this polymer as stealth-inducing material. However, the mechanism of clearance dependent on the presence of anti-PEG antibody is only partially understood. A recent study by Grenier et al. (2018) focused on the clearance rate of different PEGylated molecules, such as PLGA NPs, liposomes, and BSA, which demonstrated how even a single administration of PEG-PLGA NPs could induce the production of anti-PEG IgM and increase NP clearance by their activation of the complement system through the classical pathway. This not only reduced the circulation time of PEG-PLGA NPs after a second administration but also induced cross-reaction toward PEGylated liposomes, increasing also their clearance. However, the injection of either free PEG or PEGylated albumin was not subjected to quicker clearance even in PEG-sensitized animals, meaning that PEG is not *per se* immunogenic. This means that PEG is not *per se* capable of boosting its own clearance after chronic exposure, but it is also its disposition as an ordered pattern on the NP surface that induced the high IgM reactivity against PEG-coated NPs. Furthermore, this work demonstrated how the clearing effect of IgMs was mediated by their activation of the complement system through the classical pathway. Conversely, the PC of PEGylated NPs showed a decreased amount of complement proteins involved in alternative and lectin-dependent pathway and were enriched in apolipoprotein E (ApoE). This study is of importance since it demonstrates that NP architecture, itself, is a critical component in their immunogenicity.

However, despite PEG demonstrating to reduce the NP uptake by the MPC clearing cells, in some instances, it also decreased the interactions of NPs with the cells *de facto* reducing their targeting efficiency (Suk et al., 2016), creating the so-called “PEG dilemma.” After overcoming the BBB, NPs encounter a new biological compartment. For this reason, some studies also focused on the evaluation of PEG on the NPs targeting efficiency of different CNS cell populations. Jenkins et al. demonstrated how the use of PEG to coat magnetic NPs reduced their uptake from CNS immune cells (the microglia) but at the same time also reducing the uptake from neurons (Jenkins et al., 2016).

**Figure 3:**
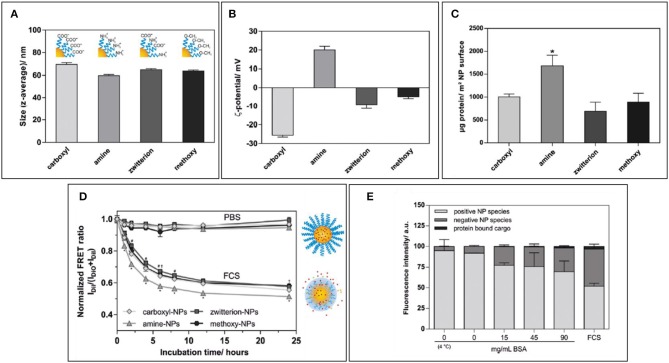
**(A,B)** Size and zeta potential of NPs coated with amino-polyethylene glycol (PEG), carboxy-PEG, both (zwitterionic), or with methoxy-PEG. **(C)** Protein amount per NP surface among differently coated NPs. **(D)** Normalized FRET ratio used to quantify Dil leakage from different NP formulations over time during incubation with FCS. **(E)** Quantification of fluorescence on agarose gel in which amino-PEG NPs were run after incubation with increasing amounts of BSA or FCS. Figure reproduced with permission from Abstiens et al. (2019) with modifications. *p < 0.05.

The density of PEG has also an important repercussion on NP stability, as highlighted by a recent study by Seneca et al. (2018). The enrichment of some important stealthy proteins such as apolipoprotein A1 and clusterin occurred at even a low concentration of surface PEGylation, demonstrating how the use of PEG, itself, could result in the presence of important proteins that could improve NP pharmacokinetics. However, the overall protein adsorption, colloidal stability, and lower uptake by macrophages still required higher percentages of polymer to be achieved, demonstrating how a good NPs surface coverage is paramount to achieve optimal NP stealthiness. Similarly, Abstienze et al. analyzed the assembly of PC on different PEG-polylactic acid (PEG-PLA) NPs (Abstiens et al., 2019). These NPs presented on their surfaces different combinations of PEG terminal groups: positively charged amines, negatively charged carboxyl groups, a combination of the two (zwitterionic), or non-charged methoxy groups. As expected, the amine-coated NPs presented faster and higher protein absorption, and lower stability in serum. Carboxyl-PEG and methoxy-PEG NPs showed lower PC assembly. Interestingly, the lowest protein absorption was observed in zwitterionic-PEG NPs. The higher interactions with methoxy-PEG NPs are understandable if we consider that proteins can interact with non-charged groups through hydrophobic interaction. Zwitterionic particles showed also the highest stability in serum. It is interesting to observe that the increase in protein absorption on NPs is not only a cause of destabilization but can also cause hydrophobic cargo leakage, even with stable particles, as demonstrated by Dil and DiO release measured by FRET. This can be explained by a two-step process: first, the partition of the hydrophobic drug from the particle core to the proteins, followed by dynamic interchange of proteins on the NP surface leading to more drug leeching (Figure 3).

This concept of PEG capable of manipulating the PC emerges in another study by Chen et al. (2019), in which the different composition and coating of siRNA-loaded solid lipid NPs (SLNs) could modulate the composition of PC, with either positive or negative repercussions on the cellular uptake rate and efficacy. In this study, particles were functionalized with different ratios of either C14-PEG or C18-PEG. Notably, SLNs coated with C14-PEG had their uptake and transfection efficiently increased by the presence of FBS during incubation. This could be due to the easier removal of C14 compared to C18 by the proteins composing the PC. The removal of PEG from the surface of SLNs could enhance the interactions with the cells and thus their uptake and consequent gene silencing effect. The most abundant proteins composing the PC were found to be either ApoA1 or ApoA2, with only one formulation having albumin as the most relevant protein. Interestingly, after incubating the different formulations with ApoA1, it was found that there was no substantial effect of this protein on NP efficacy, and conversely, only after incubating the C14-PEG-coated SLNs with ApoE was the efficacy enhanced.

Despite the many studies that focused on the definition of minimal PEG density on NPs to guarantee good stealthiness, even at the highest PEG density, a small amount of proteins is able to penetrate the polymeric coating, coming into contact with the NP surface and bypassing the PEG itself. A recent work by Li et al. (2019) demonstrated how the use of crosslinked PEG endowed AuNPs with enhanced colloidal stability, reduced protein adsorption, and reduced macrophage uptake while still resulting as non-cytotoxic. Hyperbranched polyglycerol (hbPG) has also been tested as a potential stealth-inducing material (Weber et al., 2019). This material demonstrated the ability to reduce the overall amount of assembled proteins on the surface of functionalized liposomes, and the composition of PC assembled on hbPG-coated NPs was remarkably like the one of PEGylated liposomes. However, the uptake by macrophages *in vitro* of hbPG-functionalized liposomes was substantially higher than that of the PEGylated particles.

PEG remains a staple in the development of long circulating nanomaterials due to its long-established use and the presence of FDA-approved PEGylated NP formulations. Nevertheless, the development of new materials to overcome PEG limitations offer more, and perhaps better, options for new nanovectors.

Poly-phosphoesthers (PPEs) have recently gained a lot of attention as a novel stealth-inducing polymer thanks to its ease of synthesis, biocompatibility, and biodegradability, with high efficiency in prolonging NP circulation (Schöttler et al., 2016). These features make PPEs a biodegradable alternative to PEG, which is still characterized by low degradability and the formation of toxic byproducts. In a recent study, four different polymers were used to non-covalently coat the NPs through adsorption (Müller et al., 2017). Surfactants with a longer hydrophilic side were found to bind in lower number on each NP, most likely because of higher steric hindrance. Furthermore, all the PPE-based surfactants were able to substantially reduce the amount of human serum albumin and complete serum bound to the NPs, and partially prevent NP aggregation in serum. Remarkably, SDS-PAGE analysis showed how all the polymers in use were able to modify the PC composition, reducing greatly the amount of bound IgG and albumin compared to bare NPs, and instead attract high amounts of ApoA1 and clusterin, both believed to enhance the stealth properties of NPs. Finally, NPs coated with the PPE surfactant with the higher binding constant were also phagocytized to a much lower extent than bare NPs by macrophages.

Zwitterionic polymers have recently gained attention as a novel strategy to produce stealthy NPs (Estephan et al., 2011). In a recent study (Loiola et al., 2019), silane NPs have been dually functionalized with 3-(dimethyl (3-(trimethoxysilyl) propyl)-ammonium) propane-1-sulfonate (SBS, a zwitterionic moiety) and with either amino groups, carboxyl groups, or thiol groups. The coating with SBS was able to substantially decrease the absorption of BSA on the surface of NPs, as demonstrated by SDS analysis, and stabilize them in a biological environment, compared with chemically reactive NPs. Furthermore, zwitterionic coating prevented the interaction of NPs with RBCs, thus, preventing hemolysis. Interestingly, the introduction of the zwitterion moiety also greatly reduced their interactions with viruses, bacteria, and mammalian cells, mediated by amino groups, carboxyl groups, or thiols. These phenomena can be explained by the high hydrophilicity of SBS, which induced the formation of a thermodynamically favored hydration layer that covers other biologically active ionized functional groups (steric hindrance). Another family of zwitterionic polymers that gained attention as stealth-inducing material is represented by sulfobetaines. In a recent study, Affonso de Oliveira et al. (2018) demonstrated that silica NPs functionalized with reactive primary amines onto their surface had markedly reduced PC formation and markedly lowered cytotoxicity and hemolytic activity when modified also with the zwitterionic sulfobetaines groups. Another example is offered in a recent study by Ye et al. (2016). In this work, starch-based polymeric nanosized micelles were functionalized with hydrophobic and super-hydrophilic (ammonium and sulfate) groups in order to induce the efficient self-assembly of micelles, which presented on their surface a high density of both positive and negative charges. The zwitterionic micelles showed remarkable stability in the presence of BSA and FBS and showed reduced protein adsorption compared to the non-zwitterionic formulations. Furthermore, the zwitterionic micelles were not cytotoxic or hemolytic, and demonstrated the ability to avoid phagocytosis by macrophages *in vitro* and were not able to activate them. Finally, *in vivo* analysis demonstrated how this novel stealth formulation was able to substantially increase the circulation time of DOXO compared to the free drug. The authors argue that the stealth proprieties of the NPs in study arise from the high hydration of their surface, which in turn decreases the NP–protein interactions. However, the *in vitro* studies on macrophage uptake of these nanovectors was performed in the absence of FBS, and thus with no PC onto the micelles. This makes the assessment of the relevance of the PC in modulating NP–cell interactions difficult to assess *per se*. In this perspective, the comparison of NPs having or not a PC is necessary to elucidate whether the stealth-inducing mechanism of zwitterionic polymers is indeed mediated by the reduced PC or just by repulsive forces against the cell membranes.

Although the stealth-inducing materials can provide a great benefit for NP pharmacokinetics and improve their tumor accumulation through passive targeting, the uptake of NPs by the target tissue is often slow and non-specific. In order to overcome this, the use of active targeting moieties on the surface of NPs has gained attention. These moieties represent a wide swath of chemically heterogeneous molecules that range from small compounds such as folic acid, to big proteins such as antibodies and soluble proteins. They are selected in order to bind specific receptors present on the target cells, thus increasing the endocytic uptake of NPs. However, since these moieties are exposed onto the surface of NPs, they also modify the interface identity and its interaction with the biological environment they are in. Thus, their effect on the PC assembly and composition must be considered since it adds another layer of complexity to the design of NPs. The use of different moieties has been extensively discussed in other works and goes beyond the scope of the present paper (Blanco et al., 2015; Parodi et al., 2015). Only a few studies have focused on this issue. One example of this innovative approach is a recent study by Su et al. (2018). In this study, a wide array of combinations of AuNPs functionalized with different lengths of PEG loading cyclic RGD peptides (which binds to integrin α_n_β_4_) has been employed. By analyzing the uptake by tumor cells and macrophages, the authors established that the optimal formulation was represented by PEG2000 NPs coated with cRGD (at a density of 75%). The uptake by macrophages was particularly reduced, however, by NPs coated with PEG10000. For all formulations, the presence of serum during the incubation substantially hindered NP uptake in all cases, although with some variability among formulations. Finally, the presence of PC showed overall homogenous presence of proteins with sporadic fluctuations in the total amount but with overall comparable profiles. Although this study does not provide a conclusive solution on how to optimize targeting while avoiding phagocytosis, the use of a wide range of combinations and the considerations of multiple variables in NP design offers an interesting insight on PC-informed NP design approach. Furthermore, it was also demonstrated that NPs in the presence of serum are internalized by cells by different endocytic/phagocytic pathways compared to the serum-free conditions, especially shifting the uptake by macrophages from phagocytosis to clathrin-dependent endocytosis.

Other studies have investigated the effect of PC on the targeting efficiency of actively targeted NP formulations. One example of this is offered by Salvati et al. (2013) in a study on fluorescent silica NPs functionalized with transferrin through a PEG linker. In this work, the specific targeting capabilities of NPs toward the transferrin receptor were substantially impaired by the exposure of cells to NPs in the presence of FBS, since NPs were similarly endocytosed in cells expressing the target receptor or knock-downs using siRNA transfections. This kind of studies are of critical importance to understand if a candidate-targeting moiety could be a viable option to make the search for new active targeting strategies better biologically informed. Similar results showing the detrimental effects of PC on active targeting were obtained on chitosan NPs targeted with an aptamer directed against the glycoprotein MUC1, which is overexpressed in certain colorectal cancers (CRC) (Varnamkhasti et al., 2015).

Nevertheless, in a different study by Dai et al. (2015), layer-by-layer polymer PMA nanocapsules or NPs were actively targeted with a monoclonal antibody against huA33, another molecule often overexpressed in (CRC). Interestingly, despite the differences in PC composition among nanocapsules and NPs, the active targeting of these formulations was retained even in the presence of high amounts of FCS.

These discrepancies in the results make our understanding of the effect of PC onto the targeting efficiency of NPs quite fragmented and incomplete. The factors that determine the outcome of this complex interaction are poorly understood, and more studies on the exact interactions occurring between PC and any given active targeting moieties are necessary to shed light on this issue.

The specific proprieties of a material, however, are not the only features determining their stealthiness. Formulative variables such as the procedure for NP coating, the polymer conformation, and its surface density are critical parameters in order to achieve the optimal stealth effect. This concept was explored in a recent work by Coty et al. (2017). In this research, the authors investigated the potential of dextran coated poly-(isobutyl cyanoacrylate) NPs to activate the complement system, and through which pathways, based on the density and chemistry of the surface dextran. The results showed that protein accessibility to the NP surface was a major contributor to complement activation, especially through the classical pathway. Thus, NPs with lower coating density were the ones that triggered the complement system the most. However, also the accessibility of complement proteins to terminal sugar groups or hydroxyl groups enhanced complement activation through the lectin and alternative pathway, respectively. The use of longer and more densely packed dextran did not cause any relevant amount of complement activation through any pathway. These results somewhat consolidate what was previously established for PEG (Zhou et al., 2018).

### Biomimetic Approaches

#### Cell Membrane Coating

One of the most well-established biomimetic approaches to increase NP circulation time is coating them with cell membranes. The use of entire cell membranes allows the NP surface to *de facto* recapitulate the features of the cells used as a substrate and especially the complex array of membrane proteins they normally have. Membrane proteins give the cells a biological identity both as part of the same organism (the so-called “self” recognition) and define their intercellular and molecular interactions, providing an inherent initial biologic identity. However, when thinking about the normal path of a NP formulation after injection, the first environment it meets is blood. Thus, using cell membranes and membrane proteins from circulating cells appears an ideal solution in order to bestow NPs with long circulation by camouflaging them as biological components normally present. The use of these cells types allows the NPs to interact with the proteins present in the blood similar to how the cells do, and thus, by separating the NPs from their surroundings, they can provide not only colloidal stability but also avoid opsonization and the production of anti-NP immunoglobulins that could result in quick NPs clearance and potentially C activation-related pseudo allergy.

Following this concept, a wide array of formulations was produced by using a handful of cellular sources: red blood cells (RBCs), platelets, leukocytes, and cell-derived exosomes. RBC-coated NPs are among the first biomimetic formulations employed (Ding et al., 2015; Rao et al., 2016; Xia et al., 2019). The use of RBCs as source for cell membranes is particularly convenient since these cells do not have nuclei or most organelles, so they can be reduced to small vesicles upon simple disruption. Moreover, they are abundant in the blood and can be purified by simple centrifugation; they present on their surface an array of proteins such as CD47, which are considered “don't eat me” signals. CD47 binds to SIRPα on macrophages and activates an intracellular signaling cascade within the macrophages that ultimately leads to the inhibition of phagocytosis. This immune-elusive mechanism is believed to be responsible for the improved tolerability and circulation time of RBC-coated NPs (Ye et al., 2019). However, because RBCs are characterized only by their ability to circulate for a long time and normally do not associate with any specific tissue, they only improve the NP pharmacokinetics only through passive targeting, thus limiting their utility.

In order to confer the NPs also active targeting properties, the use of platelets has been considered. Indeed, platelets not only retain the simple molecular composition and long circulation time like RBCs but also possess the ability to bind to damaged blood vessels, thus providing also a rudimentary level of active targeting toward cardiovascular damage (Evangelopoulos et al., 2018). A more advanced approach resulted in the use of leukocytes as a starting material for membrane protein extraction. Leukocytes represent an almost ideal source since they are circulating cells, have still “self” proteins, but they also normally adhere to inflamed blood vessels, extravasate in the surrounding tissue, and interact with foreign bodies. Thus, the use of leukocytes can provide active targeting for inflammation, making the use of such coating highly valuable for a wide array of applications that range from tumors to chronic inflammatory states such as sepsis IBDs (Molinaro et al., 2016, 2019; Corbo et al., 2017a; Martinez et al., 2018).

Despite the potential advantages of cell membrane coating, there are only a few studies that investigate on the PC of these still novel formulations. In fact, even if their surface composition resembles the ones of cells, their size is still much reduced, and their very production could have some impact on the delicate composition of the membranes and membrane proteins they are coated with. This could occur by losing some components of the original cell membranes, such as glycosylation, or partially desaturate the membrane proteins, or again by displaying proteins with the right orientation on the NP surface.

**Figure 4:**
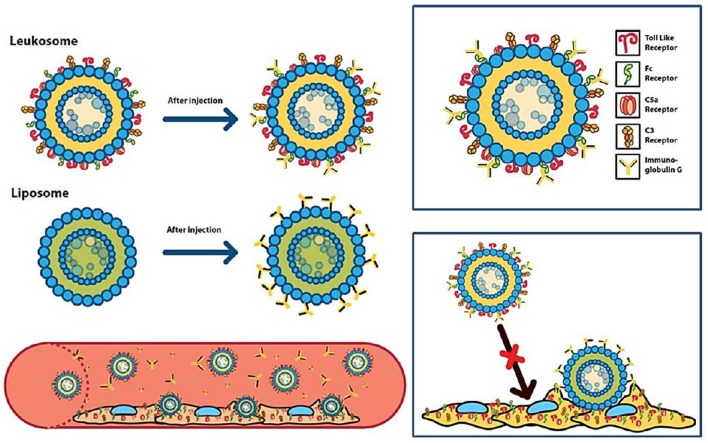
Schematic representation of the differential immunoglobulin-binding capabilities of liposomes and leukosomes. Figure reproduced with permission from Corbo et al. (2017b). Copyright (2017) American Chemical Society.

Only a few studies have focused on the investigation of the PC of biomimetic nanosystems. An example among these is the recent work from Corbo et al. (2017b). In this study, control liposomes were compared with lipid nanovesicles in which phospholipid bilayer presented leukocyte membrane proteins. These biomimetic vesicles are defined as “leukosomes” (Figure 4). Liposomes or leukosomes were injected IV in BALB/c mice, and the blood was harvested either after 10 min or 1 h after injection. Remarkably, leukosomes demonstrated a lower amount of bound proteins at both time points. Confirming past studies on different NPs, the protein composing the PC had an IP <6, and a MW <60 kDa. Interestingly, the composition of PC between liposomes, although similar, presented some critical differences. Namely, leukosome PC presented higher amounts of clusterin, which, as discussed, is an essential protein in determining the long circulation of injected NPs. The different composition of the protein corona was correlated with the observation of longer circulation time and lower MPS uptake of leukosomes *in vitro* and *in vivo*. Another fascinating mechanism proposed in this work relies on the presence on leukosomes of immunoglobulins receptors (FcRs) normally present on leukocytes. Theoretically, leukosomes, through these receptors, could bind circulating immunoglobulins not through their antigen-recognizing variable portion, but through the constant domains. This could present the immunoglobulins activation as primers for complement activation and opsonization by the MPC *de facto* making them beneficial for the biodistribution of leukosomes. This intriguing mechanism underlines once again the ambiguity of function of certain protein classes composing the PC, since not only their presence but also their conformation and orientation could have an important impact on the overall final impact of PC on the fate of NPs.

#### Use of Viruses and Viral Components

Viruses have been considered an inspiration in the design of drug delivery nanovectors since the inception of this field of research (Parodi et al., 2017). Viruses are, themselves, small particles able to circulate with ease in the body and reach a wide array of target tissue with high selectivity and can deliver even molecularly complex and otherwise unstable cargoes, especially nucleic acids, with high transduction efficiency. However, a few data have been produced about the specific surface proprieties of viruses and how they interact with proteins in biologic fluids. A recent work by Berardi et al. (2019) focused on comparing the formation of PC on either standard polystyrene NPs (PS-NPs) with the PC assembled on cowpea mosaic virus (CPMV) NPs and bluetongue virus (BTV) NPs. Notably, CPMV NP mobility in an agarose gel electrophoresis was not influenced by high concentrations of either BSA, FBS, pepsin, or mucin, demonstrating the lack of a substantial PC forming on these NPs and increasing their weight. This is quite a different profile from PS-NPs which showed a much-decreased mobility in agarose that decreased with increasing protein concentration. BTV NPs instead, demonstrated aggregation at high proteins levels. These data not only show that CPMV NPs were stable in a wide array of biological environments and that not all viral NPs have suitable proprieties for drug delivery. Furthermore, CPMV NPs showed superior mucus penetration compared to PS-NPs and BTV NPs. The favorable stealth behavior of CPMV NPs was attributed by the small size and viral surface with both positive and negative charged at high density, making it an ideal zwitterionic coating. However, the use of viral NPs is severely hindered by their potential immunogenicity. Although their clearance does not appear to be mediated by PC assembly, their circulation *in vivo* has been demonstrated to prime the production of anti-virus immunoglobulin that triggers their quick clearance from circulation. To overcome this critical hurdle, more studies on the molecular composition of viral surface could be useful to distinguish among the components responsible for their stealthiness while removing the potential immunogenic motifs.

Another study by Xu et al. (2016) generated artificial viral NPs (AVNs) composed of a core of AuNPs coated with a phospholipid bilayer modified with the ganglioside GM3 as an active targeting moiety for CD169 normally expressed by APCs. The studied formulation presented different percentages of 1,2-dioleoyl-sn-glycero-3-phospho-L-serine (DOPS) in their surface composition and were either 35 or 80 nm in diameter. Interestingly, the assembly of PC was substantially lower in larger particles, as demonstrated by the smaller increase in hydrodynamic diameter shown by DLS analysis. Furthermore, the higher the percentage of DOPS, the lower the ζ of the NPs became, and the more proteins bound to the AVN surface. Therefore, larger particles with lower DOPS amount demonstrated higher stability in the presence of serum. In turn, this increased PC formation resulted in a sensible decrease in GM3 targeting efficiency toward CD169 *in vitro*. After IM injection, AVNs demonstrated efficient targeting of lymph nodes toward the peripheral portion of the lymph nodes and co-localized with CD169 immunostaining. This study, although does not offer a thorough insight in the PC relevance on AVN fate, still offers important elements on the importance of NP size and composition in modulating the PC thickness.

#### Protein Corona Manipulation

The new understanding of PC as an unavoidable feature of NP interactions with biological systems and the potential beneficial role of dis-opsonizing proteins in enhancing NP pharmacokinetics sparked the interest in manipulating the PC composition instead of creating PC-avoiding materials.

In a recent work, Almalik et al. compared chitosan NPs (CS-NPs) coated with different stealth-inducing biologic materials: alginate or hyaluronic acid (Alg-CS-NPs and HA-CS-NPs, respectively) (Almalik et al., 2017). The use of either coating substantially increased the size of NPs, modulated the otherwise positive surface charge of CS-NPs to a more favorable negative ζ, increased their stability in the presence of serum, and substantially reduced the formation of PC. Interestingly, the use of either Alg or HA conditioned the assembly of the PC. More specifically, non-coated or Alg-CS-NPs presented a wide array of proteins on their PC that were involved in immunogenicity. On the contrary, the use of HA substantially reduced the presence of these proteins and was even shown to provide PC with potentially beneficial action such as α-1-acid glycoprotein (AGP) and inter-α-trypsin inhibitor heavy chain H4, proteins associated with anti-inflammatory functions.

This is a good example of how the intrinsic proprieties of the coating material can make NPs less immunogenic *de facto* bestowing them with stealth-like proprieties and increasing their safety. However, the author proposes even a more radical approach: after the establishment of specific anti-inflammatory proprieties as constituents of the PC, the next logical step would be the use of these proteins, themselves, as a coating material. Following this logic, plasma proteins have been used in order to improve the pharmacokinetics of otherwise quickly cleared biologic vectors. One example of this approach is offered by Gulati et al. (2018). In this study, tobacco mosaic virus (TMC), a prototypical rod-shaped drug delivery vector, was covalently coated either with PEG or with PEG-conjugated serum albumin (SA) in order to reduce the generation of anti-TMC antibodies. Different coating densities and PEG lengths were tested. Interestingly, although coating efficiency was proportional to PEG/TMC ratio, longer PEG linkers led to lower coating efficiency on the TMC NPs, due to higher surface of the NPs occupied by each PEG molecule, both for PEG alone and PEG-SA. Further analysis showed how the use of more dense coating caused a substantial decrease in TMC molecules by antibodies. This analysis also evidenced how the use of shorter linkers to produce SA coating prevented the recognition of PEG, itself, by anti-PEG antibodies.

The *in vivo* testing, however, demonstrated that after repeated injection of all the SA-TMC NPs, anti-TMC antibodies were still generated and detectable in the blood. Interestingly, these antibodies were not as efficient in detecting SA-TMC NPs, and no anti-SA antibodies were generated (which could have led to systemic auto-immune reactions). This phenomenon could be explained by the differential processing of TMCs and SA after phagocytosis by macrophages, as shown by confocal microscopy imaging after *in vitro* incubation of SA-TMC onto RAW 264.7 cells. Indeed, it was demonstrated, in fact, that after phagocytosis, TMC NPs were trafficked toward the lysosomal compartment (and so toward antigen processing), while the conjugated SA followed a quick recycling path back to the plasma membrane. This study is a remarkable example of how the proper assessment of the biological fate of stealth nanosystem can give important insights in the actual mechanism of immunogenicity of a drug delivery vector.

Another interesting example of how the biologically informed design of a nanomaterial can achieve a great improvement in its interaction with the biological environment was given by Magro et al. (2019). In this study, 10-nm iron oxide NPs (IONPs) were produced. These particles showed good stability in water and were characterized by a highly positive ζ, caused by the surface presence of Fe (III) ions. These IONPs were loaded with the antibiotic oxytetracycline (OTC) on their surface. After administering them to female zebra fish, their protein-binding profile was analyzed. Remarkably, IONPs and OTC–IONPs were mostly associated with ApoA1, an apolipoprotein considered to be stealth inducing (Schöttler et al., 2016). However, the sole binding of these proteins is not enough to achieve a stealthy behavior; adsorbed proteins should also maintain their natural conformation, avoiding a possible immunogenic denaturation. Remarkably, IONPs had a size, shape, and surface charge organization that could efficiently interact with ApoA1 dimers, trimers, and tetramers in their native conformations through electrostatic interactions. The biodistribution analysis of these NPs showed that once dispersed in zebrafish farming water, they were up taken by the animals especially through intestinal absorption, and that they mostly targeted the animals' ovaries. This is in good agreement with the ApoA1 fate, since this protein is highly endocytosed by ovaries in this species.

Following the idea of some commercially available albumin-based drug delivery systems (e.g., Abraxane), Li et al. formulated a novel formulation of PLGA NPs coated with PEG presenting on its surface-reactive maleimide moieties (Li et al., 2018). The rationale of this design is the high reactivity and relative selectivity of maleimide with albumin by covalently binding the cysteine-34 on this protein. This, in turn, would provide a stabilized irreversible albumin-based PC that could not only enhance NP pharmacokinetics but also targeting. The functionalization with maleimide, in fact, substantially increased total protein absorption and enriched the amount of albumin onto the NP surface compared to plain PLGA NPs and PEG-PLGA NPs. Furthermore, PLGA-PEG-Mal NPs demonstrated a more efficient uptake by cancer cells that was dependent on BSA concentration during the incubation and by the cell expression of proteins involved in the uptake of albumin, itself (e.g., gp-60 and SPARC) (Sleep, 2015). In *in vivo* experiments, PLGA-PEG-Mal NPs had a circulation half-life comparable to PLGA-PEG, but after multiple administration, the production of anti-PEG immunoglobulins was lower than for PLGA-PEG NPs. Furthermore, PLGA-PEG-MAL demonstrated more efficient targeting in tumor-bearing mice. This is most likely, thanks to a higher uptake of albumin by tumor cells.

Another recent work by Oh et al. (2018) further expands and improves this model. In this study, the authors coated mesoporous silica NPs (MSNPs) with a recombinant protein composed by the fusion of HER2-binding affibody (Afb) with glutathione-S-transferase (GST)29 through an extra linker, forming protein corona-shielded MSNPs (PCSNs). This protein was bound to the surface of NPs through a covalent bond with the glutathione (GSH) molecules used to functionalize the NP surfaces. This protein was stably bound to the MSNP surface and maintained their functional conformation and proper orientation. The PC assembly and composition on PCSNs was compared to the one of PEG-MSNPs and GSH-MSNPs. Remarkably, the protein corona shielding decreased the overall amount of adsorbed proteins, and the PC contained much less complement and coagulation proteins compared to the other groups. Furthermore, the PCSNPs also provided improved targeting to HER2-expressing cells, thanks to the function protein active-targeting capabilities, while being endocytosed much less by macrophages, even when compared to PEG-MSNPs. Furthermore, in efficacy *in vivo* experiments in tumor-bearing mice, PCSNPs loaded with the chemotherapeutic camptothecin were able to efficiently target the tumor while accumulating much less into clearing organs (e.g., liver, spleen, and kidneys) and had higher therapeutic efficacy than PEGylated NPs. This is a true demonstration of how the advanced manipulation of the PC can provide new stealth and active targeted nanovectors that show improved profile even when compared to the polymers considered as gold standard for NP long circulation.

Other studies have achieved similar results using non-covalent NP coating with serum proteins. A study by Yeo et al. (2018) demonstrated how AuNRs could be stabilized through their incubation in mouse serum. Furthermore, this pro-formed PC allowed efficient loading of photodynamic therapy (PDT) enabling molecule Ce6. These NPs showed, after injection, an increased tumor accumulation in a murine xenograft model of cancer compared to bare AuNRs and demonstrated high therapeutic efficacy. The tumor accumulation was attributed not only to the EPR effect enabled by increase in NP diameter after PC assembly but also by the high concentration on the PC of albumin and apolipoproteins, thought to be able to work as targeting agents for gp60 and LDL receptors, respectively.

Another interesting approach that allows for the manipulation of PC corona formation *in vitro* and *in vivo* is the strategy of molecular imprinting (Komiyama et al., 2003). This strategy formulates NPs whose components are attached covalently or non-covalently to specific molecules. After the formation of NPs, the said protein is removed by the NPs through chemical or enzymatic means, thus, leaving a template of the protein onto the new NPs and, thus, forming an “artificial receptor” able to bind to the protein present in solution. This concept was applied to molecularly imprinted polymeric nanogels (MIP-NGs) formulated in the presence of HSA (Takeuchi et al., 2017). These particles, after the removal of albumin, showed much higher affinity toward HSA immobilized on an SPR probe and were also able to bind strongly soluble fluorescent HSA as demonstrated by FRET analysis. After IV injections MIP-NGs showed substantial longer blood half-life and much lower liver accumulation compared to the non-imprinted particles. FRET analysis performed using fluorescent HSA *in vivo* confirmed that even in circulation, MIP-NPs bind mostly HSA, thus, *de facto* modulating the PC composition toward a more well-tolerated and long circulation profile.

Techniques for the manipulation of PC can also offer innovative techniques to overcome notoriously difficult biological barriers. The archetype of such hard-to-tackle obstacle is represented by the BBB, which prevents brain targeting using traditional drugs due to reduced diffusion and active extrusion of active molecules from the CNS. This challenge was undertaken by Zhang et al. (2019) in a recent work. In this study, novel biomimetic liposomes, functionalized with a peptide derived from Aβ amyloid (SP) and loaded with DOXO (SP-Lipo-DOXO) for the treatment of glioblastoma, were tested. The rationale of using SP resides in its ability to bind apolipoproteins such as ApoA1, ApoE, and ApoJ after IV injection. These proteins not only work as disopsonins but also as *in situ* recruited targeting moieties, since they are able to bind to specific receptors present on both the BBB and often expressed by glioblastoma cells, themselves. This approach allows for the use of the own patients' circulating proteins as targeting, thus, avoiding complicated synthetic steps in the formulations and avoiding the use of non-self-proteins that could be immunogenic. LP-Lipo-DOXO was able to efficiently bind apolipoproteins both in *in vitro* and *in vivo* conditions. This resulted in increasing CNS targeting *in vivo*, improved DOXO delivery to glioblastomas, and an increase in survival compared to the non-functionalized liposomes. Furthermore, the NPs did not show an increased immunogenicity compared to plain liposomes, resulting in the formulation being safe even after multiple administrations.

## Conclusions and Future Perspectives

In this review, the difficulties in the PC investigation were evidenced by presenting all the known NPs and experimental variables that can alter the PC composition. Despite the complexity of this field of study, still many studies rely on simple *in vitro* setups for PC assembly induction and on non-very informative techniques (i.e., SDS-PAGE). Moreover, there is not a standard workflow that could be used to obtain more systematic PC information that could be more easily comparable among different studies. Although the use of *in vivo* models in conjunction with proteomic analysis can greatly enhance the quality and amount of the obtainable information, the use of animals exclusively for PC assessment could be quite demanding in terms of logistics and funding, not mentioning the ethical implications. Furthermore, the precise indication of all experimental settings and the implementation of standardized experimental guidelines could further harmonize the PC characterization. Despite the high interest in the study of PC relevance in NP platform development, proteins are not the only biological molecules interacting with nanovectors. Recently, some studies shed light on the binding of lipids on NPs, in particular, lipoproteins present in plasma.

In one of these works (Müller et al., 2018), polystyrene NPs were incubated with different ratios of different purified lipoproteins and apolipoproteins. The binding constants and stoichiometry of these molecules were measured using ITC. The binding affinity increased with the decrease in lipoprotein density; *vice versa*, larger (and less dense) lipoproteins interact in lower numbers with NPs. Interestingly, lipoproteins were shown to interact with NPs more strongly than apolipoproteins, indicating the role of lipids, themselves, in determining the lipoprotein affinity. Furthermore, this study evidenced that there is a significant fraction of lipoproteins disintegrating on the NP surface, leading to direct covering of NPs by lipids. This was further reinforced by the high NP retention of cholesterol after multiple centrifugation. Importantly, it was demonstrated that after incubating NPs with pure lipoproteins, their uptake by RAW264.7 macrophages was highly reduced. Taken together, all these data underline the importance of including not only proteins in the study of NP coronae but also the potential utility in exploiting lipoproteins as a novel clocking mechanism to increase NP circulation time, thus, offering a new strategy to improve their targeting capabilities. This innovative concept is considered a broader view on NPs' biological interactions with molecules, and often defined as “biomolecular corona,” since it includes proteins, lipids, sugars, and other metabolites (Capriotti et al., 2019). Moreover, the variation of PC composition between healthy and sick individuals, depending even on the specific disease they are affecting from, could be used to induce PC assembly and the assessment of the patient health state depending on its composition. Some recent studies demonstrated the potential of this approach for diagnostic purposes (Zheng et al., 2015; Caputo et al., 2017).

Furthermore, most current studies on PC composition focus on the study of the so-called “hard corona” composed of tightly bound proteins, and only a few efforts are put in the development and application of techniques that can separate NPs with even loosely bound proteins on their surface (Pederzoli et al., 2018). Thus, the composition and biological relevance of the soft corona is still largely unknown. One of the few studies shedding light on this difficult topic was performed by Weber et al. In this study, the PC composition of PEGylated polystyrene NPs was analyzed after either centrifugation or asymmetric flow field-flow fractionation (AF4) (Weber et al., 2018). This technique relies on very low shear stress that can, thus, preserve most of the soft PC on NP surface. Remarkably, the composition of hard corona from both techniques was similar, so the different composition of the PC between the two techniques was attributed to the presence of the soft corona. Interestingly, the AF4-isolated PC had, in percentage, much more immunoglobulins and much less ApoA1 and clusterin. However, despite this radically different composition, the cellular uptake by HeLa cells was not substantially different among the particles separated using the two techniques, perhaps due to the dissociation of soft corona after dilution of NPs before the treatment. The biological significance of the soft corona is still elusive, and more efforts are required in order to establish how (and if) the soft PC has repercussions on NP behavior in circulation.

Regarding which design paradigm for NPs holds the greatest potential, both the use of synthetic and biomimetic approaches for the fabrication of stealthy NPs bring with themselves their own sets of advantages and caveats. In fact, the use of stealth-inducing polymers often reveals itself as a double edged sword, especially in the context of developing NPs for solid tumor treatment: the use of highly hydrophilic, slightly negatively charged polymers provides substantial escape from opsonization and quick clearance by MPCs; however, these same proprieties can severely hinder the efficiency of the interactions with the target cells once NPs reach the desired tissues, by reducing the tissue penetration because of the higher hydrodynamic diameter, and looser associations with cell membrane because of the repulsive electrostatic charges. Conversely, the use of active targeting moieties and cell-penetrating peptides substantially enhances the NP uptake, but at the same time increases the formation of PC, facilitating their opsonization and, thus, reducing greatly their plasma half-life. This formulative dilemma has been undertaken by several groups, and many elegant solutions spawned by the combinations of stealth-inducing and uptake-enhancing materials on the same NPs (Juang et al., 2019). These innovative formulations can switch their behavior depending on external stimuli: they can either respond to their chemical and biological milieu, or they can be “activated” by external stimuli. Therefore, NPs would behave “stealthily” during systemic circulation, allowing for improved EPR-mediated tumor accumulation, and instead present their active targeting and cell-penetrating moieties after reaching the tumor milieu.

The rise of the biomimetic philosophy emerged in response to these limitations: compared to the time consuming, complicated chemical synthesis, and the research of intelligent yet biocompatible materials, the use of biologic molecules appears as an ideal solution. In fact, biological materials are biocompatible and provide a staggering variety of functions and behaviors that can be exploited to improve NP formulations. The use of cell-derived membranes provides NPs with the same proprieties of the cell source in terms of circulation, tissue targeting, and cell-to-cell interactions. Furthermore, the creation of an artificial protein corona bestows a pre-determined biological identity to the NPs, thus allowing for the “hacking” of the immune system, itself. In some instances, entire cells have been used as drug delivery vectors, exploiting completely their natural capabilities. However, this approach is also limited by some critical hurdles that have yet to be assessed. In particular, the use of biomaterials can raise some question regarding their immunogenicity and safety (e.g., the use of non-autologous proteins or viral components); furthermore, their complex structure and composition still poses severe problems regarding the reproducibility and reliability of their proprieties. Following these considerations, none of these approaches to stealthy NP formulations are ideal and can achieve ideal behavior and at the same time satisfy the need for scalability that the clinical praxis demands. Perhaps the combination of the finely tuned chemical synthesis and of the biocompatible and versatile biologics could converge and generate a new, holistic paradigm of stealth NPs design.

The potential coating of NPs with an artificial PC has also an amazing potential for increasing the circulation time of nanomaterials, and even providing natural active targeting moieties, using purified (and perhaps even recombinant) proteins, and thus avoiding the complications of using entire membrane proteins or a wide array of membrane proteins. This approach could allow to produce finely tuned highly reproducible biomimetic formulations. Another fascinating approach could be the use of patient-derived purified disopsonins to create a personalized coating for each patient on chemically reactive NPs, resolving any potential issue of immunogenicity that haunts many biomimetic formulations. The future nanomaterials could even be composed entirely of disopsonin proteins, not different from the already currently available albumin-based nanovector Abraxane™. However, these new horizons heavily depend on the bottleneck of our limited understanding of which proteins composing the PC are the most critical in improving NP biodistribution.

Regarding this limiting factor, many studies on the PC are in several cases contradictory. For example, immunoglobulins are often associated with poor NP stability and quick clearance from circulation. However, some studies also demonstrated how immunoglobulin are negatively associated with NP uptake from macrophages and even how they can work as disopsonins by binding to the FcR on NPs presenting this receptor. Thus, immunoglobulins enhance NP clearance only if they can bind to specific epitopes on the NP surface, thus, activating and making NPs visible to immune cells. If Igs, however, bind non-specifically or not through their antigen-recognizing domains to the NP surface, it is likely that they work as any other protein of the same size and surface charge, thus losing their immunological relevance.

In conclusion, our understanding of the PC composition, relevance, and manipulation has substantially expanded in the last years. However, the experimental difficulties in its characterization and its over-simplified interpretation have led to only partial and, in some cases, potentially skewed understanding of the role of different protein classes that are present in the PC. In this perspective, it is necessary to develop in the future harmonized techniques for the study of PC assembly and its analysis. These techniques should cover both *in vitro* and *in vivo* investigation, with attention to their translational potential. Furthermore, extensive study of currently approved nanovectors in clinical setting could provide invaluable information on *in vivo* human PC. The analysis of PC should take great advantage from the “omics” techniques, which provide unprecedented amount of information on such complex samples. Finally, an extensive and standardized panel of the PC assembly conditions and results should be provided, in order to make the studies more easily comparable and consistent. This panel should include already well-established biological tests such as the uptake and activation by macrophages and complement activation. All these changes are complex and require the collaboration of all the major experts on the PC field in order to unlock the staggering complexity of PC overcoming the contradictory nature of the current results and thus create a coherent, comprehensive, and predictive model describing the relevance of PC in NP stability, biodistribution, and safety.

## Author Contributions

RR: writing the original draft with support from SC. PC and SP: revising the article. MA: conception and design.

### Conflict of Interest

The authors declare that the research was conducted in the absence of any commercial or financial relationships that could be construed as a potential conflict of interest.

## Abbreviations

AF4: asymmetric flow field-flow fractionation
Afb: affibody
AuNPs: gold nanoparticles
AuNRs: gold nanorods
BBB: blood–brain barrier
BTV: bluetongue virus
CD: circular dichroism
CPMV: cowpea mosaic virus
CRC: colorectal cancer
DLS: dynamic light scattering
EPR: enhanced permeability and retention effect
FBS: fetal bovine serum
FcR: Fc receptor
GIT: gastrointestinal tract
GSH: glutathione
GST: glutathione-S-transferase
HPC: hard protein corona
HuPC: human protein corona
IBD: inflammatory bowel disease
IP: isoelectric point
ITC: isothermal titration calorimetry
IV: intravenous
MPC: mononuclear phagocytic system
MS/MS: tandem mass spectrometry
MuPC: murine protein corona
NP: nanoparticle
OTC: oxytetracycline
PC: protein corona
PDI: polydispersity index
PDT: photodynamic therapy
PEG: polyethylene glycol
PGMA: poly-(glycidyl methacrylate)
PPE: polyphosphoesters
RBC: red blood cell
RGD: arginylglycylaspartic acid
SA: serum albumin
SBS: 3-(dimethyl (3-(trimethoxysilyl) propyl)-ammonium) propane-1-sulfonate
SLN: solid lipid nanoparticle
SPC: soft protein corona
SPR: surface plasmonic resonance
TEM: transmission electron microscopy
TMV: tobacco mosaic virus
ζ: zeta potential.
